# Opposite effects of visual and auditory word-likeness on activity in the visual word form area

**DOI:** 10.3389/fnhum.2013.00491

**Published:** 2013-08-29

**Authors:** Philipp Ludersdorfer, Matthias Schurz, Fabio Richlan, Martin Kronbichler, Heinz Wimmer

**Affiliations:** ^1^Centre for Neurocognitive Research and Department of Psychology, University of SalzburgSalzburg, Austria; ^2^Neuroscience Institute, Christian-Doppler-Clinic, Paracelsus Medical University SalzburgSalzburg, Austria

**Keywords:** fMRI, neuroimaging, one-back task, word-likeness, word processing, VWFA, orthographic representations

## Abstract

The present fMRI study investigated the effects of word-likeness of visual and auditory stimuli on activity along the ventral visual stream. In the context of a one-back task, we presented visual and auditory words, pseudowords, and artificial stimuli (i.e., false-fonts and reversed-speech, respectively). Main findings were regionally specific effects of word-likeness on activation in a left ventral occipitotemporal region corresponding to the classic localization of the Visual Word Form Area (VWFA). Specifically, we found an inverse word-likeness effect for the visual stimuli in the form of decreased activation for words compared to pseudowords which, in turn, elicited decreased activation compared to the artificial stimuli. For the auditory stimuli, we found positive word-likeness effects as both words and pseudowords elicited more activation than the artificial stimuli. This resulted from a marked deactivation in response to the artificial stimuli and no such deactivation for words and pseudowords. We suggest that the opposite effects of visual and auditory word-likeness on VWFA activation can be explained by assuming the involvement of visual orthographic memory representations. For the visual stimuli, these representations reduce the coding effort as a function of word-likeness. This results in highest activation to the artificial stimuli and least activation to words for which corresponding representations exist. The positive auditory word-likeness effects may result from activation of orthographic information associated with the auditory words and pseudowords. The view that the VWFA has a primarily visual function is supported by our findings of high activation to the visual artificial stimuli (which have no phonological or semantic associations) and deactivation to the auditory artificial stimuli. According to the phenomenon of cross-modal sensory suppression such deactivations during demanding auditory processing are expected in visual regions.

## Introduction

Since the advent of neuroimaging a multitude of studies has shown that the left ventral occipitotemporal cortex (vOT) plays a crucial role in reading (for reviews see Jobard et al., 2003; Vigneau et al., 2006; Price, 2012). Specific attention has been given to a region at around *y* = −58 (MNI space) in a series of studies by L. Cohen, S. Dehaene and collaborators who reasoned that it hosts abstract representations of letter strings and, hence, referred to it as Visual Word Form Area (VWFA; Cohen et al., 2000, 2002; Dehaene et al., 2001). However, different from its name, the VWFA was assumed to be only involved in sub-lexical processing of legal letter sequences and not in whole-word recognition (Dehaene et al., 2002). This VWFA hypothesis stimulated critical debate (Price and Devlin, 2003, 2011) and a large number of imaging studies investigated the functional properties of this region. Dehaene et al. (2005) extended the original VWFA hypothesis by proposing hierarchical stages of visual word recognition along the left ventral visual stream from detection of letter fragments in V2 to recognition of recurring letter strings and short words in anterior vOT (*y* = −48) with in-between stages of letter and letter sequence coding. Support for the hierarchical model was provided by Vinckier et al. (2007) who presented words and letter strings of decreasing similarity to words (frequent quadrigrams, frequent bigrams, frequent letters, infrequent letters and false-fonts) and found increasing response selectivity along the ventral stream with absent differentiation in posterior regions and a marked differentiation in anterior regions. Specifically, in anterior regions (including the VWFA) activation was highest for words and lowest for strings of infrequent letters and false-fonts.

Our research group contributed to the VWFA debate by proposing that vOT regions may not only be involved in sub-lexical coding as assumed by the VWFA hypothesis, but also provide visual-orthographic whole-word recognition, similar to other anterior vOT regions which are engaged by recognition of visual objects or faces (see Kronbichler et al., 2004). This proposal was based on our finding that a parametric increase in word frequency from pseudowords to high frequency words was accompanied by a systematic decrease of VWFA activation. Further studies tried to control for the confound between orthographic familiarity and phonological/semantic familiarity. Therefore, we compared familiar to unfamiliar spellings (i.e., pseudohomophones) of the same phonological words (e.g., TAXI vs. TAKSI) in a phonological lexical decision task (“Does XXX sound like a word?” with YES to both familiar and unfamiliar spellings) and found decreased VWFA activation to the familiar spellings (Kronbichler et al., 2007, 2009). Similar inverse effects of orthographic familiarity on VWFA activation (i.e., words < pseudohomophones) were found in several other studies (Bruno et al., 2008; van der Mark et al., 2009; Twomey et al., 2011).

An interesting extension of inverse orthographic familiarity effects on vOT activation was recently provided by Wang et al. (2011) using a one-back repetition recognition task. The authors investigated the effects of word-likeness with stimuli ranging from familiar Chinese characters to unfamiliar artificial characters. They found higher vOT activation to artificial Chinese characters compared to pseudo-characters (composed of two semantic and/or phonetic components) which, in turn, elicited higher activation compared to existing Chinese characters. Interestingly, this inverse relation between word-likeness and activation was present from posterior (*y* = −80) to anterior (*y* = −48) regions of the left ventral visual stream.

The inverse effect of word-likeness on brain activation found by Wang et al. stands in contrast to the findings of Vinckier et al. who found the exact opposite pattern. To further investigate this discrepancy, a first goal of the present study was to investigate effects of word-likeness of visual stimuli along the ventral visual stream, especially in left vOT. Therefore, similar to Wang et al., we presented familiar (German) words, pseudowords and unfamiliar artificial stimuli (false-font strings) in the context of a one-back task. In cognitive terms, words can be considered to be more familiar than pseudowords due to the presence of orthographic word representations (with associations to phonology and meaning) and pseudowords are more familiar than false-font strings due the presence of representations for letter identities and for recurrent letter sequences within words. The question was whether the large extension of the inverse word-likeness effect of Wang et al. would also be found with alphabetic stimuli. Specifically unexpected would be a posterior extension of the decreased activation for familiar words compared to pseudowords. This finding would imply that visual recognition for words is already present in posterior vOT regions. In our previous studies—mentioned above—decreased activation to familiar words compared to unfamiliar letter strings (pseudohomophones and pseudowords) typically only emerged in anterior vOT regions and only these were assumed to be involved in orthographic whole-word recognition.

The main novel aspect of the present study was the extension of our stimuli set to auditory stimuli also varying in word-likeness (i.e., familiar words, pseudowords and unfamiliar reversed-speech stimuli). The inclusion of auditory stimuli is of interest in relation to the controversy around the question whether the VWFA—as implied by its name—is indeed a region primarily (or even exclusively) engaged by visual processes. In support of this assumption, Dehaene et al. (2002) showed that, compared to a rest baseline, the VWFA exhibited marked activation in response to visual but not to auditory words. However, a study by Price et al. (2003) found increased activation to auditory words compared to an auditory control condition. From this and similar findings (e.g., Booth et al., 2002). Price and Devlin (2003) inferred that the VWFA potentially is a “polymodal” region. More recently, a study by Reich et al. (2011)—similar to a previous by Buchel et al. (1998)—found that the VWFA was engaged by Braille reading in congenitally blind subjects. Based on these findings, Dehaene and Cohen (2011) recently referred to the VWFA as a “meta-modal” reading area. However, this conclusion may be premature. In congenitally blind people, regions dedicated to visual processing may be taken over by other sensory information such as touch. In sighted readers, the VWFA may still be a region primarily engaged by visual processing.

Specifically interesting for the VWFA's modality-specificity is the phenomenon of “cross-modal sensory suppression” (Laurienti et al., 2002; Mozolic et al., 2008). According to this, demanding auditory processing can be expected to result in negative activation (compared to rest baseline) in regions primarily engaged by visual processes. Indeed, a recent study by Yoncheva et al. (2010) provided support for negative activation of the VWFA to auditory stimulation. This study presented a sequence of two tone patterns which had to be matched. This tone-matching task served as control condition for a rhyme matching task in which two auditory words were presented. In accordance with cross-modal sensory suppression, the tone-matching task resulted in negative activation (compared to rest baseline) in bilateral occipital and OT regions including the VWFA. The auditory words presented for rhyme matching also resulted in deactivation of bilateral OT regions with the exception of the VWFA where activation was increased (close to rest) compared to tone-matching. A possible explanation is that, during the presentation of auditory words, the VWFA was exempt from cross-modal sensory suppression due to activation of visual word representations associated with the auditory words. This interpretation would be in line with behavioral studies which found rhyming judgments to be prone to misleading “orthographic intrusions” (Seidenberg and Tanenhaus, 1979).

The present auditory stimuli differed markedly from those of Yoncheva et al. as we presented only single auditory items (words, pseudowords) and the present one-back task may be less prone to activating associated orthographic word representations compared to the rhyme-matching task of Yoncheva et al. Therefore, it was of interest whether the result pattern of Yoncheva et al. will be replicated. Furthermore, a main advantage of the present study is that the activation of left vOT regions in response to the visual stimuli can be topographically related to those in response to the auditory stimuli. If the expected increased activation to the auditory words (compared to negative activation for the artificial stimuli) in vOT regions is indeed resulting from orthographic information associated with the auditory words, then this activation cluster should roughly coincide with the expected visual orthographic familiarity effect (i.e., visual words < pseudowords).

## Materials and methods

### Participants

Twenty-nine (11 female) participants aged 19–48 years (*M* = 24.3 years) took part in the experiment. All participants were German speaking university students, had normal or corrected-to-normal vision, and had no history of neurological disease or learning disability. The study was approved by the ethical committee of the University of Salzburg. All participants gave written informed consent and were paid for participation.

### Stimuli and procedure

Stimuli consisted of visually and auditorily presented words, pseudowords and modality-specific artificial stimuli (false-fonts and reversed-speech, respectively). In total, each participant viewed 40 items (36 and 4 repetitions) of each of the categories. Initially, lists of 72 items were generated for the words and pseudowords. The words were all German nouns, one half were names for tools (e.g., “Hammer”), and the other half names for animals (e.g., “Zebra”). Word length varied from four to eight letters, and from one to three syllables. Mean word frequency was 4.59 per million (based on the CELEX database; Baayen et al., 1993). The pseudowords matched the words in number of letters, number of syllables, bigram frequency, and number of orthographic neighbors. Counterbalanced across participants, one half of the words and pseudowords were presented visually and the other auditorily. Artificial visual stimuli were generated by presenting the visual words in false-fonts. Artificial auditory stimuli were constructed by time-reversing the auditory word recordings.

In the scanner, subjects performed a one-back repetition task and were instructed to press a button with their right index finger whenever they viewed two identical stimuli in succession. Responses were considered as hits when they occurred before presentation of the next stimulus. An absence of response during this period was considered as a miss. All other responses were considered as false alarms.

Auditory stimuli were spoken by a male voice and presented via MR-compatible headphones. Visual stimuli were presented in yellow on a dark grey background. The visual display was projected by a video beamer (located outside the scanner room) on a semi-transparent screen, and viewed by the participants via a mirror mounted above their heads. An MR-compatible button box was used for the participants to respond. Projection and timing of the stimuli, as well as the recording of responses, was controlled by Presentation (Neurobehavioral Systems Inc., Albany, CA, USA).

Presentation of the items was divided into two functional imaging runs, with each run containing both visual and auditory stimuli as well as 20 null-events during which a fixation cross presented in the middle of the screen. A fast event-related design was used to investigate the hemodynamic response to the different types of stimuli. The order of items and null-events within each run was determined by a genetic algorithm (Wager and Nichols, 2003). Stimulus onset asynchrony (SOA) was 3800 ms. Visual stimuli were presented for 820 ms and the average duration of the auditory words was 808 ms, ranging from 600 to 1150 ms. During presentation of auditory stimuli, as well as during the interstimulus intervals, a fixation cross was present. The fact that the SOA is not a multiple of the used TR (2000 ms) enhances the efficiency of sampling the hemodynamic response at different time points. The total duration of the functional session was approximately 20 min (10 min per run).

### Image acquisition and analysis

A 3-Tesla TRIO TIM Scanner (Siemens, Erlangen, Germany) was used for both functional and anatomical MR imaging. For the functional runs, images sensitive to blood oxygenation level dependent (BOLD) contrast were acquired with a T2^*^-weighted echo-planar imaging (EPI) sequence using a 32 channel head coil. In each run consisted of 309 functional images (Flip angle = 70°, *TR* = 2000 ms, *TE* = 30 ms, FOV = 210 mm, 64 × 64 matrix). Thirty-six descending axial slices (thickness = 3.0 mm; inter-slice gap = 0.3 mm) were acquired. In addition, for each subject a high-resolution structural scan was acquired with a T1-weighted MPRAGE sequence. The resolution of the structural image was 1 × 1 × 1.2 mm.

Preprocessing and statistical data analysis was performed using SPM8 software (http://www.fil.ion.ucl.ac.uk/spm) running in a MATLAB 7.6 environment (Mathworks Inc., Sherbon, MA, USA). Functional images were realigned, unwarped, and then co-registered to the high-resolution structural image. The structural image was normalized to the MNI T1 template image (using SPM's segmentation procedure). The resulting parameters were used for normalization of the functional images, which were resampled to isotropic 3 × 3 × 3 mm voxels and smoothed with a 6 mm full width at half maximum (FWHM) Gaussian kernel.

The functional data were high-pass filtered with a cut-off of 128 s, as removing frequencies below 1/128 Hz reduces low frequency drifts. For correction of temporal autocorrelations an AR (1) model (Friston et al., 2002), as implemented in SPM8, was used. Statistical analysis was performed within a two stage mixed effects model. On the individual level, the parameter estimates reflecting signal change for each item type vs. rest (= null events and ISIs) were calculated. Item repetitions were separately modeled as regressors of no interest. Additionally, six covariates corresponding to the movement parameters (rotations and translations) were included. The subject-specific contrast images were used for the second level (random effects) analysis, which allows generalization to the population. For all statistical comparisons we used a voxel-wise threshold of *p* < 0.001, and a cluster extent threshold of *p* < 0.05, corrected for family-wise error (FWE).

## Results

### Behavioral

Response latencies for the detected repetitions in the one-back task (see Table 1) were entered in an ANOVA with the factors modality (visual and auditory) and stimulus type (words, pseudowords, and artificial). Table 1 shows that there were longer response latencies for the auditory than the visual stimuli, *F*_(1, 28)_ = 42.73, *p* < 0.05. This is, however, not surprising since participants had to wait until the auditory stimulus presentation was completed in order to make a correct decision. Stimulus type had no reliable effect, *F*_(2, 56)_ = 1.48, *p* = 0.24, and did not interact with modality, *F*_(2, 56)_ < 1.

**Table 1:** **Means (standard deviations) of response latencies and accuracy measures (hit- and false-alarm rates) in the one-back task**.

**Measures**	**Visual**			**Auditory**		
	**Words**	**PWs**	**Artificial**	**Words**	**PWs**	**Artificial**
Response latency (ms)	811.7	770.8	847.2	967.3	965.3	1040.9
	(240.0)	(300.1)	(409.1)	(251.0)	(325.4)	(333.9)
Hit-rate (%)	91.4	87.9	80.2	95.7	91.4	90.5
	(18.0)	(20.7)	(27.0)	(15.0)	(20.4)	(21.6)
False-alarm-rate (%)	3.4	0.3	0.9	3.0	0.5	0.7
	(17.0)	(1.1)	(2.0)	(14.4)	(1.7)	(1.2)

Task accuracy was generally high as shown by the mean hit and false-alarm rates in Table 1. Across stimulus types hit rates for the visual stimuli were on average lower than those for the auditory stimuli, *F*_(1, 28)_ = 4.30, *p* < 0.05. There was neither a reliable effect of stimulus type, *F*_(2, 56)_ = 1.07, *p* = 0.35, nor an interaction between the factors, *F*_(2, 56)_ < 1. However, as shown in Table 1, for the visual artificial stimuli the mean hit rate was lower than the rate for the other stimuli (but still at about 80%). In addition, false-alarm rates were generally very low, only the words (visual and auditory) had a slightly increased rate.

### fMRI

Given our theoretical focus on ventral visual regions, especially left vOT, we limited our main fMRI analyses to anatomical regions involved in ventral visual stream processing: inferior occipital, inferior temporal, and fusiform gyrus of both hemispheres as defined by automatic anatomical labeling (AAL; Tzourio-Mazoyer et al., 2002). Results of additional whole-brain analyses are provided in the Supplementary Materials.

#### Visual stimuli

**Figure 1:**
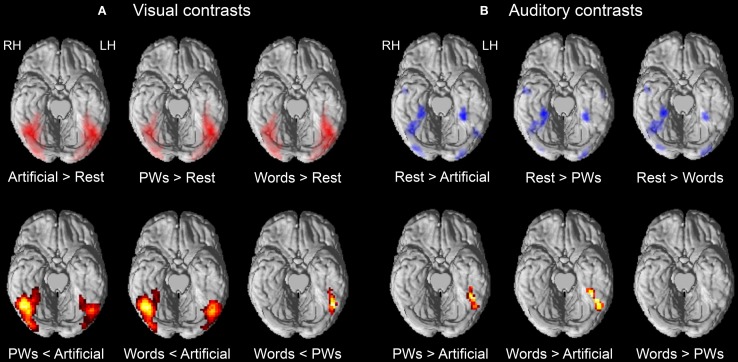
**Brain activations for visual (A) and auditory (B) contrasts of interest**. The upper row depicts contrasts against rest baseline. Positive and negative activations are depicted in red and blue, respectively. The lower row depicts results of contrasting the stimulus types with each other. Activation maps are shown on the ventral surface of a rendered cortex. All contrasts are thresholded at *p* < 0.001 voxel-wise with a cluster extent threshold of *p* < 0.05 (FWE corrected). LH, left hemisphere; RH, right hemisphere.

Initial contrasts against rest baseline showed that all three types of visual stimuli elicited widespread positive activations throughout ventral occipital and temporal regions (see upper row of Figure 1A). Of main interest were the contrasts corresponding to the expected inverse effect of word-likeness. As mentioned in the Introduction, following Wang et al. (2011) we expected reduced activation for pseudowords compared to the artificial stimuli and, following our previous findings of orthographic familiarity effects (Kronbichler et al., 2004, 2007), we expected reduced activation for words compared to pseudowords.

The findings presented in Table 2 and in the lower row of Figure 1A provide support for these expectations. The pseudowords < artificial contrast identified large bilateral clusters including occipital and OT regions with the right hemisphere cluster being more extended than the one in the left hemisphere. The maxima of the clusters were both in posterior inferior temporal regions (at around *y* = −60), but the decreased activation to pseudowords compared to artificial strings was already present in posterior occipital regions. Similar results were found for the words < artificial contrast. Furthermore, the contrast of words < pseudowords, different from the extended bilateral clusters identified by the previous contrasts, only identified a left vOT cluster. As shown in Table 2, the maximum of this cluster roughly corresponds to sites of previously found orthographic familiarity effects (Kronbichler et al., 2004, 2007). We did not identify any regions within our mask which exhibited positive word-likeness effects, that is, more activation for words or pseudowords compared to the artificial stimuli or more activation for words than pseudowords.

**Table 2:** **Visual stimuli: brain regions showing inverse word-likeness effects**.

**Contrast**	**Peak region**	***k***	**MNI coordinates**			***t***
			***X***	***Y***	***Z***	
Artificial > PWs	R Inferior Temporal	473	51	−61	−11	8.83
	L Inferior Temporal	282	−45	−64	−5	6.24
Artificial > Words	R Inferior Temporal	447	51	−61	−11	9.81
	L Inferior Temporal	249	−45	−64	−8	8.79
PWs > Words	L Inferior Temporal	85	−45	−49	−17	4.68

Peak Region is based on AAL (Tzourio-Mazoyer et al., 2002); k: cluster extent in voxel.

#### Auditory stimuli

Following the cross-modal sensory suppression phenomenon (Laurienti et al., 2002), we expected negative activation (compared to rest baseline) in response to auditory stimuli in ventral occipital and temporal regions assumed to be primarily engaged by visual processing. Additionally, following Yoncheva et al. (2010), there should be a release of deactivation for auditory words in a left vOT region corresponding to the VWFA.

Contrasts against rest baseline identified several clusters within ventral visual regions exhibiting negative activation in response to the auditory stimulus types (see upper row of Figure 1B). No cluster was identified with positive activation. Roughly similar clusters with deactivation to all auditory stimuli were found bilaterally in posterior occipital (at around *y* = −80) and medial anterior OT regions (at around *y* = −45) as well as in a right middle OT region (at around *y* = −64). In line with our expectations, in left middle OT, only the auditory artificial stimuli resulted in deactivation (at around y = −67) whereas no such deactivation was found for words and pseudowords. The contrasts between stimulus types (see Table 3 and lower row of Figure 1B) further confirmed the positive word-likeness effects in left middle OT as words and pseudowords elicited significantly more activation than the artificial stimuli. No clusters were identified with higher activation for the artificial stimuli compared to pseudowords or words and no activation differences were found between words and pseudowords.

**Table 3:** **Auditory stimuli: Brain regions showing positive word-likeness effects**.

**Contrast**	**Peak region**	***k***	**MNI coordinates**			***t***
			***X***	***Y***	***Z***	
PWs > Artificial	L Inferior Temporal	72	−39	−46	−17	4.70
Words > Artificial	L Inferior Temporal	73	−45	−58	−11	4.43

Peak Region is based on AAL (Tzourio-Mazoyer et al., 2002); k: cluster extent in voxel.

#### Region of interest analysis

**Figure 2:**
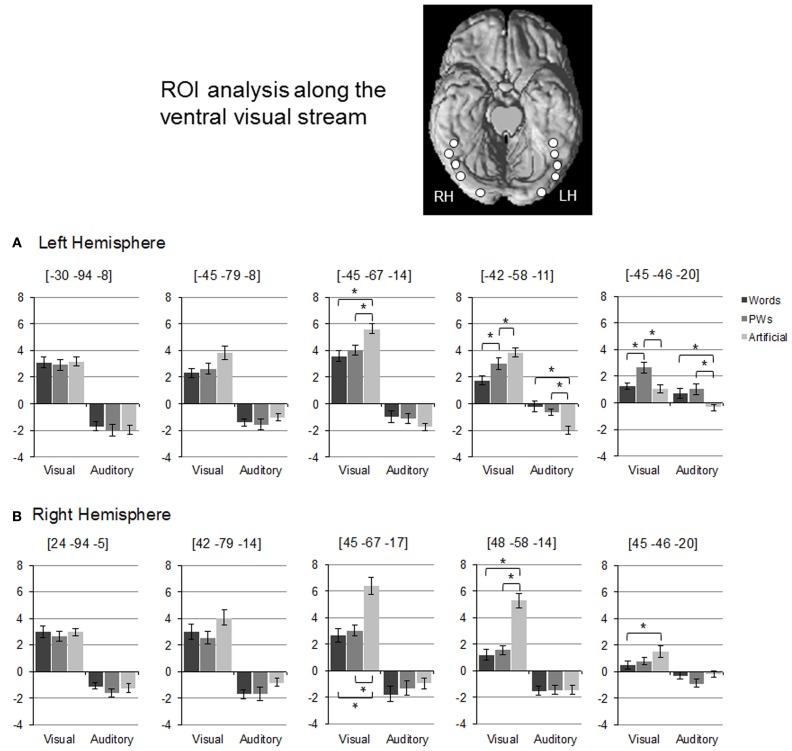
**Region of Interest Analysis: (A) and (B) depict mean brain activity estimates (given in arbitrary units) for left and right hemispheric ROIs along the ventral visual stream**. Error bars denote ± 1 standard error of mean. Approximate ROI locations are depicted on the ventral surface of a standard brain template. Asterisks denote significant differences between stimulus types (as tested with paired *t*-tests, *p* < 0.01).

For more information on changes of activation and deactivation patterns along the ventral visual stream, we relied on Region of Interest (ROI) Analysis. We selected five regions along the left ventral stream based on activation maxima of an “effects of interest” contrast (i.e. comparing all stimulus types against rest). The selected maxima approximately matched ROI locations used in Vinckier et al. (2007) and Wang et al. (2011) including one at *y* = −58 that matched classic VWFA coordinates (Cohen et al., 2000, 2002). Furthermore, to examine hemisphere differences, we selected five right hemispheric maxima, which corresponded to the left hemisphere ones on the *y*-axis. Spherical ROIs with a radius of 4 mm were built around the selected maxima (see Figure 2 for approximate ROI locations). For all ROIs, mean brain activity estimates (given in arbitrary units) were extracted. For statistical analyses, activation levels of stimulus types were compared using paired *t*-tests (*p* < 0.01).

As evident from Figure 2, there were varying activation patterns for the visual stimulus types along the left ventral stream. In the left occipital ROI (at *y* = −94), there was no difference between activation levels for the three visual stimulus types. This changed more anteriorly as in the posterior OT ROI (*y* = −67) words and pseudowords led to decreased activation compared to the artificial stimuli without differing from each other. The middle OT ROI at *y* = −58 (roughly corresponding to the classic VWFA) was the only region exhibiting an inverse visual word-likeness effect on activation levels in the form of words < pseudowords < artificial stimuli. This inverse word-likeness pattern was no longer observed in the most anterior left hemisphere ROI because activation for the artificial stimuli was much reduced (close to rest baseline). Here, activation for pseudowords was increased compared to both words and the artificial stimuli.

In the posterior ROIs of the right ventral stream, activation levels for the visual stimulus types were similar to those of the corresponding left ROIs, that is, no differences between all three stimulus types in the occipital ROI and reduced activation to words and pseudowords compared to the artificial stimuli in the posterior OT ROI. However, activation patterns differed in the two anterior right ROIs as, in contrast to the left anterior ROIs, there was no differentiation between words and pseudowords and no increased activation in response to pseudowords compared to the artificial stimuli.

Figure 2 shows that activation levels of the auditory stimuli were less differentiated than those of the visual stimuli. In the posterior ROIs of the left hemisphere, all stimulus types led to similar levels of deactivation. In the ROI corresponding to the VWFA, however, there was a positive word-likeness effect since deactivation was only present for the artificial stimuli but not for words or pseudowords which elicited reliably higher activation. This difference between words/pseudowords and the artificial stimuli was also present in the most anterior left ROI but here even the artificial stimuli did not elicit deactivation. The main finding with respect to hemisphere differences was that in the right hemispheric ROI, homologous to the VWFA, the reduced deactivation to words and pseudowords compared to the artificial stimuli was not found.

#### Conjunction analysis

**Figure 3:**
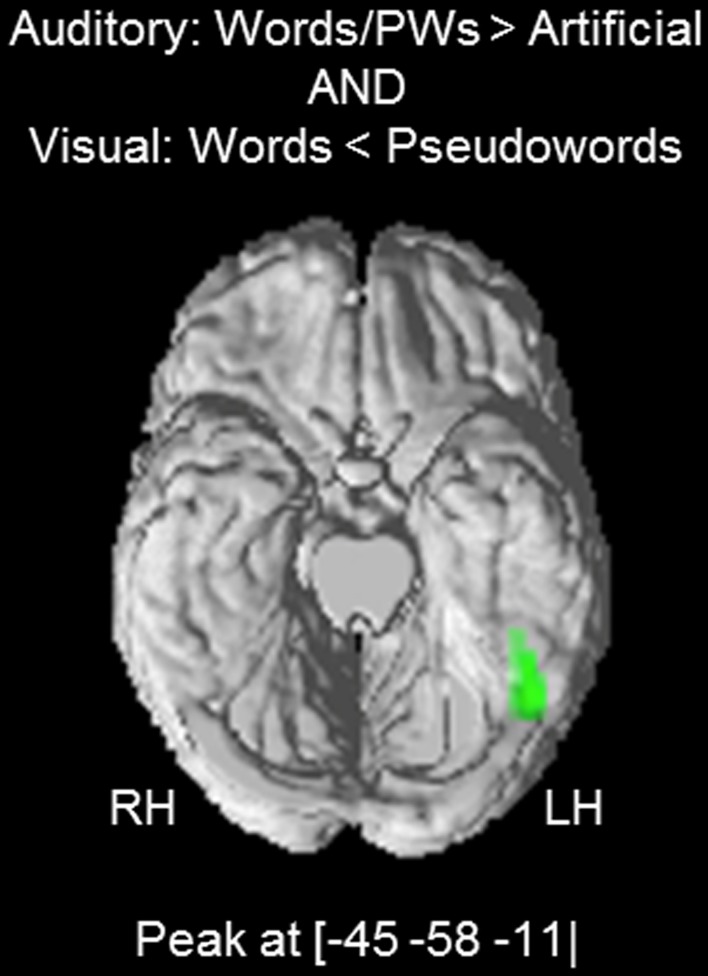
**Conjunction analysis: overlap between the auditory words/pseudowords > artificial effect and the visual words < pseudowords (*p* < 0.001 voxel-wise threshold with a cluster extent threshold of *p* < 0.05, FWE corrected)**. The activation cluster is superimposed on the ventral surface of a standard brain template.

Next, we investigated the hypothesis that the found positive word-likeness effects for the auditory stimuli (i.e., words and pseudowords > artificial) reflect activation of visual orthographic information associated with the auditory words and pseudowords. Therefore, we tested whether the auditory effects coincide with the visual words < pseudowords effect. Since there was no activation difference between auditory words and pseudowords in the previous analyses, we collapsed the data and computed an auditory words/pseudowords > artificial contrast. The resulting activation cluster had a peak at MNI coordinates [−45 −58 −11] (*t* = 4.78; cluster extent = 105 voxels). A conjunction analysis (Friston et al., 2005; Nichols et al., 2005) showed a substantial overlap between the auditory words/pseudowords > artificial and the visual words < pseudowords cluster with a peak at [−45 −58 −11] (*t* = 4.53) and a cluster extent of 44 voxels (see Figure 3).

## Discussion

### Inverse effect of word-likeness of visual stimuli on left vOT activation

We identified regionally specific differentiations between our three types of visual stimuli (i.e., words, pseudowords and artificial stimuli) along the ventral visual stream (most evident from the ROI analysis). In bilateral occipital regions (*y* = −94), all three stimulus types elicited similarly high activation. A first differentiation was found bilaterally in posterior OT ROIs (*y* = −67) where both words and pseudowords elicited decreased activation compared to the artificial stimuli and did not differ from each other. In the right hemisphere this general pattern (although with decreasing activation levels) ranged to the anterior OT ROI (*y* = −46). A further differentiation was limited to the left hemisphere. The ROI at *y* = −58 exhibited decreased activation to words compared to pseudowords in addition to reduced activation to pseudowords compared to the artificial stimuli. Reduced activation to words compared to pseudowords was also observed in the most anterior left ROI (*y* = −46), but here—different from the posterior ROIs—activation to the artificial stimuli was close to baseline.

These findings differ from those of the Chinese-based study of Wang et al. (2011) who found an inverse word-likeness effect (i.e., real characters < pseudo-characters < artificial characters) in several left hemisphere ROIs from *y* = −80 to –48. Our corresponding finding (i.e., words < pseudowords < artificial) was regionally specific to middle OT at *y* = −58 corresponding to the VWFA. Possibly, the larger extension of the contrast between Chinese real characters and pseudo-characters compared to the present words and pseudowords is due to a difference in visual familiarity. For this, one may note that the number of alphabetic letters is quite limited compared to the number of phonetic and semantic components of the Chinese script. Consequently, alphabetic words and pseudowords are visually more similar (largely sharing the same letters) compared to the Chinese characters and pseudo-characters. Based on this, Liu et al. (2008) suggested that recognition of the fine-grained Chinese characters potentially not only relies on the VWFA but also recruits posterior occipital regions bilaterally.

Importantly, the finding of reduced activation for words compared to pseudowords in a region corresponding to the VWFA is in line with the position put forward by our group that the VWFA hosts orthographic whole-word recognition units which assimilate whole letter strings (e.g., Kronbichler et al., 2004, 2007) but is also able to code letter strings of pseudowords which require sequential coding. The most direct evidence for these differing modes of processing in the VWFA is the finding by Schurz et al. (2010) who found that word length (i.e., number of letters) had an effect on VWFA activation for pseudowords but not for words. Further strong evidence for whole-word coding in the VWFA is the finding of a priming study by Glezer et al. (2009) who showed that, for real words, the exchange of a single letter from prime to target (e.g., from COAT to BOAT) led to disappearance of the priming effect, whereas the corresponding manipulation for pseudowords (e.g., from SOAT to FOAT) had little effect. More direct support for the hypothesis that the VWFA hosts orthographic representations comes from studies examining brain activation when participants have to retrieve spellings in response to auditory words (Purcell et al., 2011).

### Accounting for the opposing effects of visual word-likeness on VWFA activation in the literature

The present finding of an inverse effect of visual word-likeness on VWFA activation is just the opposite of the finding of Vinckier et al. (2007) who—as discussed in the Introduction—found a positive word-likeness effect. In the literature, there are several findings consistent with the present results. As already discussed, Wang et al. (2011) had found an inverse word-likeness effect in a more extended region along the left visual stream. Xue et al. (2006) reported higher VWFA activation to unfamiliar Korean characters compared to familiar Chinese characters and Reinke et al. (2008) found higher VWFA activation for unfamiliar Hebrew words compared to known English words. However, similar to Vinckier et al., a recent study by Szwed et al. (2011) also reported decreased VWFA activation for unfamiliar scrambled words compared to intact words, although this effect was already present in early visual areas. Importantly, these patterns were only observed in the left but not in the right visual stream.

This striking discrepancy in results between studies may find an explanation in theoretically interesting procedural differences. In contrast to the present study (and the studies reporting similar results), Vinckier et al. and Szwed et al. relied on short and rapid presentation. While the present study presented the visual stimuli for 802 ms with an SOA of 3800 ms, the presentation time of Vinckier et al. was only 100 ms with an SOA of 300 ms. One may reason that both the positive (e.g., Vinkier et al.) and the inverse word-likeness effects (present study) may reflect the same neurofunctional source, that is, the operation of whole-word recognition units (Kronbichler et al., 2004). Specifically, the fast presentation rates of Vinckier et al. and Szwed et al. may have resulted in the neuronal equivalent of the behavioral “word superiority” effect (e.g., Reicher, 1969), that is, for the letter strings of the words reduced bottom-up stimulation was still sufficient to activate whole-word codes in the VWFA which may have provided backward activation to low-level coding in posterior regions – in the case of Szwed et al. even to V1/V2. Obviously, no whole-word activation was possible for the false-font strings or the scrambled patterns resulting in diminished activation compared to words in anterior regions. The slow stimulus presentation rate of the present study together with the one-back task may have been responsible for the opposite activation pattern, that is, the inverse word-likeness effect. Specifically, the prolonged stimulus presentation allowed rather detailed coding of the artificial strings in order to set-up short-term memory representations for repetition recognition. Presumably, much less attention to the visual information was required for the letter strings of words which, in turn, provide access to the phonological words which can be easily retained for repetition recognition.

The discrepancy in the literature regarding VWFA activation for artificial visual strings also deserves a comment. To recapitulate, while Vinckier et al. and Szwed et al. found low activation levels for artificial or scrambled words, the present study—similar to other studies (e.g., Reinke et al., 2008)—found high activation. In general, these opposing results speak for the position that VWFA activation strongly depends on task setting and procedural characteristics (e.g., Starrfelt and Gerlach, 2007; Mano et al., 2013). Specifically, the high VWFA activation observed in the present study may reflect the high effort invested in encoding unfamiliar complex visual stimuli and setting-up memory representations for subsequent recognition as required by the one-back task.

One may also note that the high engagement of the VWFA for processing of the artificial strings is unexpected from the VWFA hypothesis which assumes that the region is functionally specialised for the coding of orthographic stimuli (Dehaene and Cohen, 2011). However, our findings are in line with the “neuronal recycling” hypothesis of Dehaene and Cohen (2007) which assumes that the functionally specialized VWFA emerges at a cortical location that is optimally suited for the demands of visual word processing as it is specifically tuned to dense line patterns (contours, junctions as in T L K). This expertise originally evolved for visual object recognition and is “recycled” in the course of learning to read for processing script type line patterns. In this line of reasoning, the high VWFA response to unfamiliar word-like visual patterns reflects neuronal assemblies which are critically engaged in the early phase of learning to read. We suggest that, if required, the VWFA in adult readers with a long reading history can still function as a coding system adequate for encoding and short-term representation of unfamiliar visual configurations similar to words, i.e. false-fonts.

### Positive effects of word-likeness of auditory stimuli on left vOT activation

Another main finding of the present study were the different activation patterns for the auditory stimulus types in ventral visual regions. Our expectation of general deactivations were based on the hypothesis of cross-modal sensory suppression (Laurienti et al., 2002) which states that regions primarily engaged by visual processes should exhibit negative activation (compared to rest baseline) in response to demanding auditory stimuli. In line with this expectation, deactivation to all auditory stimuli was found from posterior occipital (*y* = −94) to middle OT regions (*y* = −58) in the right hemisphere and from occipital to posterior OT regions (*y* = −67) in the left hemisphere. However, a regionally specific activation pattern emerged in a left middle OT region (*y* = −58) roughly corresponding to the VWFA. Here, marked deactivation was only present for the auditory artificial stimuli but not for words and pseudowords. This VWFA response pattern is in line with previous findings of Yoncheva et al. (2010) who had found deactivation only in response to pairs of tone-triplets but not for pairs of spoken words. The correspondence of the present finding with Yoncheva et al. is remarkable as the present auditory stimuli and our task differed markedly from Yoncheva et al. A main new finding of the present study is the substantial topographical overlap between the positive word-likeness effect for the auditory stimuli and the visual orthographic familiarity effect (i.e., words < pseudowords) in a cluster at *y* = −58. This overlap is expected when auditory words and pseudowords trigger orthographic information.

### Modality-specificity

As mentioned in the Introduction, our findings may also contribute to the question whether the VWFA—as suggested by its name—is primarily engaged by visual processes. This assumption was questioned by Price and Devlin (2003) who characterized the region as “polymodal” and more recently by Reich et al. (2011) who characterized it as “metamodal”. For this issue, the activation of the middle OT cluster (at *y* = −58 and corresponding to the VWFA) in response to the visual and auditory artificial stimuli is of specific interest. First, the high activation in response to the visual artificial stimuli cannot be related to (“polymodal”) language processes since these stimuli cannot activate phonology and/or meaning. Second, the deactivation in response to the auditory artificial stimuli can be taken to stand for the region's primarily visual role since the phenomenon of cross-modal sensory suppression (Laurienti et al., 2002) predicts that regions primarily engaged by visual processes should exhibit negative activation (compared to rest baseline) in response to demanding auditory stimuli. Taken together, these findings suggest that the VWFA belongs to brain regions primarily engaged by visual processes.

For the modality issue it is of interest that both the visual words < pseudowords and the auditory words/pseudowords > artificial effect extended to a more anterior region (*y* = −46). However, in this region, the high activation for the visual artificial and the deactivation for the auditory artificial stimuli (both present at *y* = −58) were no longer present with activation levels close to baseline. Apparently, this region is not engaged by visual orthographic processing, but is responsive to phonological processing demands. This area potentially corresponds to the lateral inferior multimodal area (Cohen et al., 2004) or a basal temporal language area (Luders et al., 1991).

## Conclusion

In the present fMRI study we investigated effects of word-likeness of visual and auditory stimuli on activation in ventral visual brain regions. In the context of a one-back task, we presented visual and auditory words, pseudowords, and artificial stimuli (false-fonts and reversed speech, respectively). The main findings were regionally specific effects of word-likeness in left vOT, in a region closely corresponding to the classic localization of the VWFA. More precisely, we observed an inverse word-likeness effect on activation for the visual stimuli (i.e. words < pseudowords < artificial stimuli) and positive word-likeness effects for the auditory stimuli (i.e. words and pseudowords > artificial stimuli). The latter resulted from a deactivation in response to the auditory artificial stimuli which was absent for words and pseudowords. We reason that the opposite effects of visual and auditory word-likeness on VWFA activation can be explained by the theoretical position that the VWFA hosts visual orthographic memory representations (Kronbichler et al., 2004, 2007). For the visual stimuli, these representations reduce the coding effort as a function of word-likeness. This results in highest activation for unfamiliar visual artificial stimuli, less activation for pseudowords and lowest activation for familiar words for which corresponding orthographic representations exist. For the auditory stimuli, higher activation for words and pseudowords compared to the artificial stimuli may result from activation of orthographic information associated with auditory words and pseudowords. The observed activation levels in response to the visual and auditory artificial stimuli also contribute to the dispute around the modality-specificity of the VWFA. First, we found high VWFA activation for the visual artificial stimuli. This cannot be explained by assuming that the VWFA is involved in general language processing since the artificial stimuli do not have phonological or semantic associations. Second, we found marked deactivation for the auditory artificial stimuli. According to the phenomenon of cross-modal sensory suppression (Laurienti et al., 2002) such deactivations during demanding auditory processing are found in visual regions. Taken together, these results speak for a primarily visual role of the VWFA.

### Conflict of interest statement

The authors declare that the research was conducted in the absence of any commercial or financial relationships that could be construed as a potential conflict of interest.
