# Supplementary material for: Opposite effects of visual and auditory word-likeness on activity in the visual word form area
Source: Front Hum Neurosci. 2013 Aug 29;7:491. doi: 10.3389/fnhum.2013.00491 (PMC3756304; doi:10.3389/fnhum.2013.00491)
Supplement: Supplementary file 1 [file DataSheet1.PDF]

SUPPLEMENTARY MATERIAL

Opposite Effects of Visual and Auditory Word-likeness on Activity in the Visual Word Form Area

Philipp Ludersdorfer[1], Matthias Schurz[1], Fabio Richlan[1], Martin Kronbichler[1,2], & Heinz Wimmer[1]

[1] Centre for Neurocognitive Research and Department of Psychology, University of Salzburg, Austria
[2] Neuroscience Institute, Christian-Doppler-Clinic, Paracelsus Medical University Salzburg, Austria

Correspondence should be addressed to P.L. (philipp.ludersdorfer@sbg.ac.at)

1. Supplementary Results

1.1. fMRI whole-brain analysis

Additionally, to the restricted fMRI analysis presented in the main text, we performed whole-brain analysis for the contrasts between visual and auditory stimulus types. All comparisons were performed using a voxelwise threshold of $p < .001$ and an additional cluster extent threshold of $p < .05$, FWE corrected.

1.1.1. Visual stimuli

Table S1 presents the results of contrasting the visual stimuli (words, pseudowords, and artificial) directly with each other. In the activation maps in Figure S1, red indicates more activation for the unfamiliar (inverse word-likeness effects) and blue indicates more activation for familiar stimuli (positive word-likeness effects).

Inverse word-likeness effects were found in numerous brain regions. More activation for the visual artificial stimuli than words and pseudowords was identified in large occipito-temporo-parietal clusters – including primary visual sensory regions, ventral and dorsal stream) as well as middle and inferior frontal regions in both hemispheres (Figure S1-A and B, respectively). More activation for pseudowords than words was found in a vOT region and an inferior frontal/precentral region (Figure S1-C).

With respect to positive word-likeness effects, more activation for words and pseudowords than for the artificial stimuli was found in several regions and included posterior superior temporal (STG) and supramarginal (SMG) regions as well as – in the case of words - an angular (AG) region (see Figure S1-A and B). An AG region was also found to exhibit higher activation for words than pseudowords (Figure S1-C).

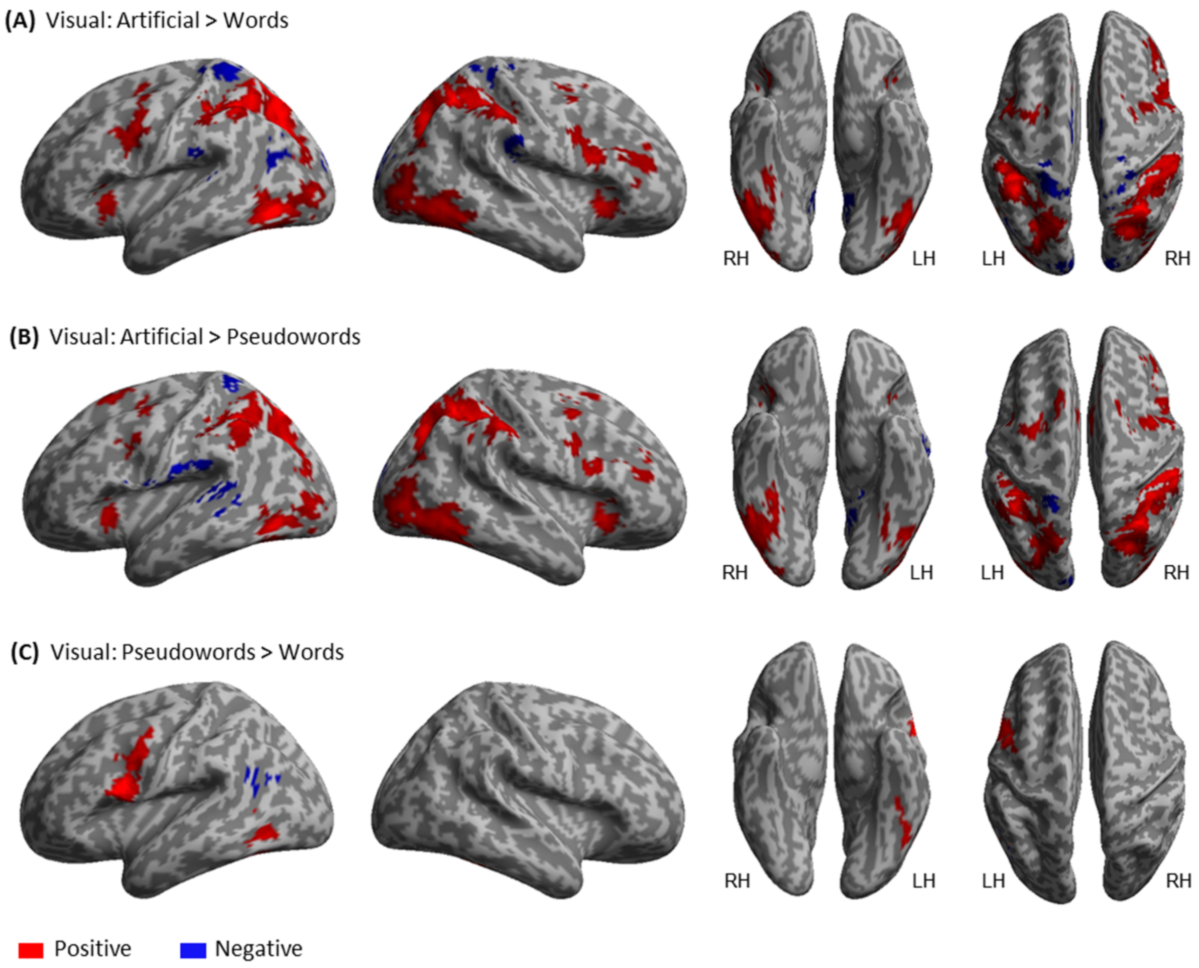

Figure S1. Contrasts between the visual stimuli (words, pseudowords, and artificial). Activation clusters are superimposed on inflated brain surfaces. For all contrasts we used a threshold of $p < .001$ at the voxel level and an additional cluster extent threshold of $p < .05$, corrected for FWE. Colors (blue represents familiarity and red inverse familiarity effects on brain activation) only depict approximate effect size (for detailed description see Table 1).

Table S1.
Brain regions activated in the visual contrasts. Clusters are sorted according to peak t value.

Region	H	k	MNI coordinates			t
			X	Y	Z	
Artificial > Words						
Middle Occipital (Inferior Parietal, Inferior Temporal, Fusiform)	R	1664	30	-67	28	9.94
Inferior Parietal (Middle Occipital, Inferior Temporal, Fusiform)	L	1472	-42	-43	43	9.09
Precentral (Inferior Frontal)	L	202	-45	5	34	8.27
Superior Medial Frontal (SMA)	R	351	6	17	46	6.74
Insula (Inferior Frontal)	R	136	33	26	-5	6.39
Inferior Frontal (Middle Frontal, Precentral)	R	370	51	11	28	6.25
Insula (Inferior Frontal)	L	115	-30	20	4	5.90
Precentral (Middle and Superior Frontal)	L	78	-24	-4	52	5.45
Middle and Superior Frontal (Precentral)	R	79	30	-1	52	4.56
Words > Artificial						
Superior Occipital (Middle Occipital, Cuneus)	L	103	-9	-97	16	5.90
Postcentral (Superior Parietal, Precuneus)	L	173	-21	-43	64	5.88
Cuneus (Superior Occipital)	R	76	15	-94	19	5.39
Precuneus (Cingulum)	L	217	-9	-55	19	4.83
Postcentral (Precuneus)	R	128	15	-49	58	4.74
Cingulum (Supplementary Motor Area)	L	136	-6	-4	40	4.72
Supramarginal	R	66	51	-31	25	4.55
Middle Temporal (Angular)	L	53	-48	-67	19	4.26
Superior Temporal (Supramarginal)	L	41	-60	-31	16	3.87
Artificial > Pseudowords						
Middle Occipital (Inferior Parietal, Inferior Temporal, Fusiform)	R	1679	51	-61	-11	8.83
Inferior Parietal (Middle Occipital, Superior Parietal, Inferior Occipital, Fusiform)	L	1236	-18	-67	55	6.80
Superior Medial Frontal (SMA, Cingulum)	L	360	6	14	49	6.03
Insula (Inferior Orbito-frontal)	R	128	30	26	-2	5.77
Cingulum	R	40	6	5	25	5.34
Inferior Frontal (Precentral)	R	177	51	11	28	5.25
Superior Frontal (Middle Frontal)	L	65	-21	17	61	5.17
Precentral (Middle and Superior Frontal)	L	100	-24	-4	52	5.12
Insula	L	79	-30	20	4	5.01
Middle and Superior Frontal	R	118	42	2	52	4.92
Precentral (Inferior Frontal)	L	46	-45	5	37	4.75
Middle and Inferior Frontal	L	67	45	38	22	4.45
Pseudowords > Artificial						
Middle Temporal (Superior Temporal, Postcentral, Supramarginal)	L	211	-60	-19	19	5.54
Superior Parietal (Postcentral)	L	49	-21	-43	67	4.96
Precuneus (Calcarine)	L	92	-12	-49	7	4.45
Pseudowords > Words						
Precentral (Inferior Frontal)	L	337	-45	2	31	7.08
Inferior Temporal (Middle Temporal, Fusiform)	L	102	-45	-49	-17	4.68
Words >Pseudowords						
Angular	L	41	-51	-58	22	3.83

Region: Anatomical label corresponding to cluster peak (according to Tzourio-Mazoyer et al., 2002). Regions to which cluster extends are given in parentheses; H: hemisphere of peak; k: cluster extent in voxel.

1.1.2. Auditory stimuli

Results for contrasting the auditory stimuli with each other are presented in Table S2. The corresponding activation maps are given in Figure S2 (word-likeness effects are color-coded analogously to the visual stimuli). Here, we expected an inverse word-likeness effect in speech processing regions and positive word-likeness effects in left vOT regions.

As can be seen in Figure S2, inverse familiarity effects in regions related to speech processing were identified in the artificial > words contrast (left and right STG) and the pseudowords > words contrast (left inferior frontal and right STG). Positive familiarity effects, i.e. more activation for words and pseudowords than the artificial stimuli, were found in left vOT. In addition, the word > pseudowords contrast identified an angular region.

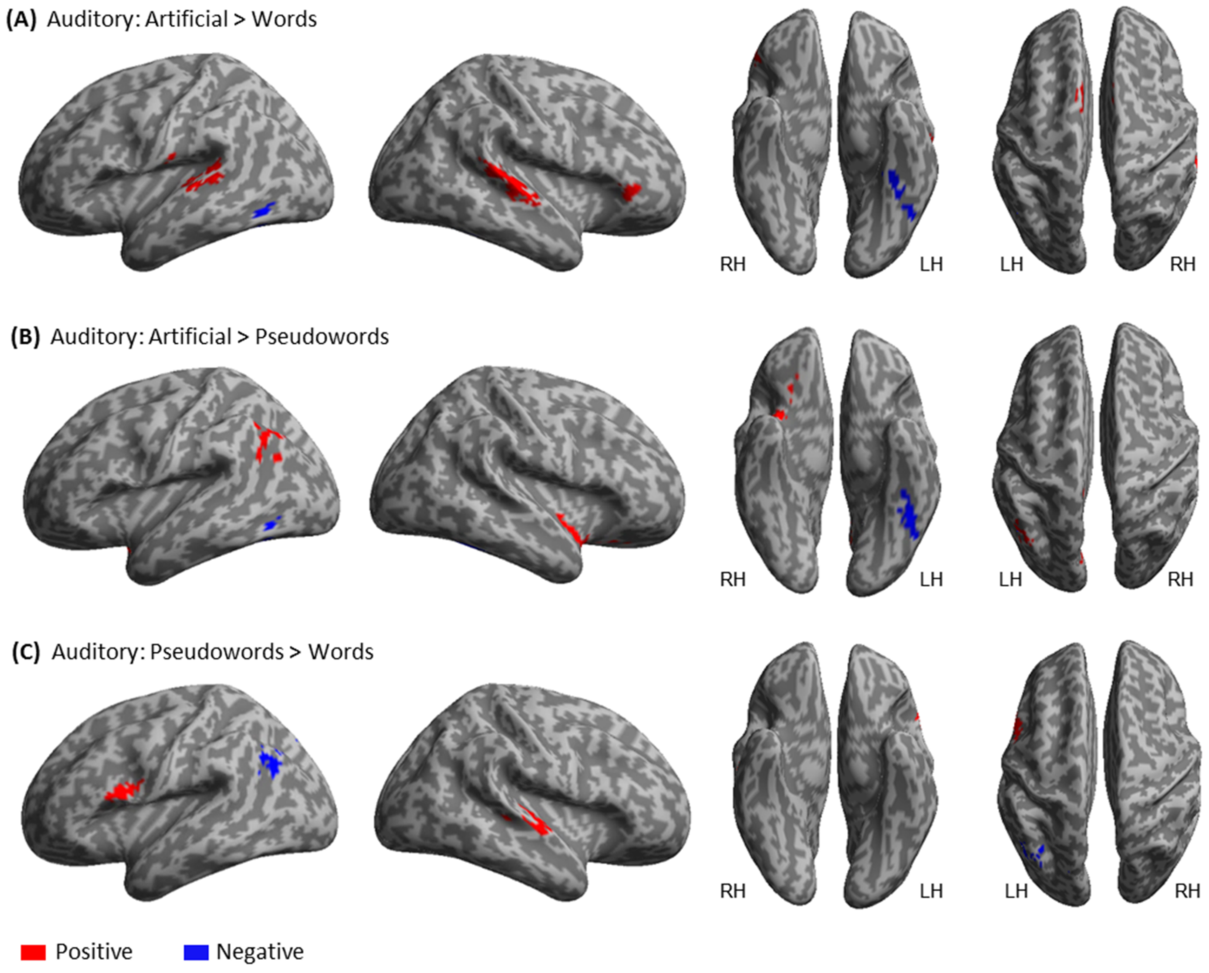

Figure S2. Contrasts between the auditory stimuli (words, pseudowords, and artificial). Activation clusters are superimposed on inflated brain surfaces. For all contrasts we used a threshold of $p < .001$ at the voxel level and an additional cluster extent threshold of $p < .05$, corrected for FWE. Color (blue represents familiarity and red inverse familiarity effects on brain activation) only depict approximate effect size (for detailed description see Table 2).

Table S2.
Brain regions activated in the auditory contrasts. Clusters are sorted according to peak t value.

Region	H	k	MNI coordinates			t
			X	Y	Z	
Artificial > Words						
Superior and Middle Temporal	R	188	63	-16	-2	5.69
SMA	R	97	3	11	64	4.80
Superior and Middle Temporal	L	117	-63	-25	4	4.41
Inferior Frontal	R	50	48	32	-2	4.03
Words > Artificial						
Inferior Temporal (Fusiform)	L	80	-45	-58	-11	4.43
Artificial > Pseudowords						
Inferior Frontal (Temporal Pole, Insula)	R	94	33	5	-17	4.95
Precunues (Cuneus, Cingulum)	L/R	91	0	-46	31	4.64
Angular (Inferior Parietal)	L	79	-48	-64	25	4.48
Pseudowords > Artificial						
Fusiform (Inferior Temporal)	L	77	-39	-46	-17	4.70
Pseudowords > Words						
Inferior Frontal (Precentral)	L	62	-51	8	16	4.51
Superior Temporal (Heschl)	R	78	63	-13	-2	4.02
Words >Pseudowords						
Angular	L	89	-48	-64	28	4.16

Region: Anatomical label corresponding to cluster peak (according to Tzourio-Mazoyer et al., 2002). Regions to which cluster extends are given in parentheses; H: hemisphere of peak; k: cluster extent in voxel.
